# Apelin Inhibits Angiotensin II-Induced Atrial Fibrosis and Atrial Fibrillation via TGF-β1/Smad2/α-SMA Pathway

**DOI:** 10.3389/fphys.2020.583570

**Published:** 2020-11-19

**Authors:** Wenkui Lv, Ling Zhang, Xinchun Cheng, Hongli Wang, Wen Qin, Xianhui Zhou, Baopeng Tang

**Affiliations:** ^1^Heart Failure Department, The First Affiliated Hospital of Xinjiang Medical University, Urumqi, China; ^2^Xinjiang Key Laboratory of Cardiac Electrophysiology and Cardiac Remodeling, The First Affiliated Hospital of Xinjiang Medical University, Urumqi, China; ^3^Department of Pacing and Electrophysiological, The First Affiliated Hospital of Xinjiang Medical University, Urumqi, China; ^4^Geriatrics Center, The People’s Hospital of Xinjiang Uygur Autonomous Region, Urumqi, China; ^5^Department of Histology and Embryology, School of Basic Medical Science, Xinjiang Medical University, Urumqi, China

**Keywords:** atrial fibrillation, apelin, angiotensin II, fibrosis, AF

## Abstract

**Background:**

Angiotensin II (Ang II) could promote the development of atrial fibrosis in atrial fibrillation (AF). Apelin can inhibit the occurrence of myocardial fibrosis. However, the effect of apelin on Ang II-induced atrial fibrosis and subsequent AF still remains unknown.

**Objective:**

In the present study, we examined the effect of apelin on the suppression of atrial fibrosis and subsequent AF, and investigated its underlying mechanisms.

**Methods:**

Sprague-Dawley rats were treated for 2 weeks with Ang II (1080 μg/kg/24 h) and apelin-13 (140 μg/kg/24 h) using implantable mini-pumps. The incidence of AF induced by atrial pacing was determined. Atrial electrophysiological mapping was recorded by a 32-electrode microelectrode array. Blood was collected to measure the levels of Ang II and apelin. Atrial tissue samples were preserved to assess the pathohistological changes, DDR2 and α-SMA co-staining were performed, and the protein expression of Smad2 phosphorylation was evaluated.

**Results:**

Apelin significantly inhibited Ang II-induced atrial fibrosis (HE:1.45 ± 0.11 vs 6.12 ± 0.16, *P* < 0.001; Masson:1.49 ± 0.25 vs 8.15 ± 0.23, *P* < 0.001; Picrosirius Red:1.98 ± 0.64 vs 9.59 ± 0.56, *P* < 0.001, respectively) and decreased the vulnerability of AF (inducibility of AF: *z* = −4.40, *P* < 0.001; total AF duration: *z* = −4.349, *P* < 0.001). Left atrial epicardial mapping studies demonstrated preservation of atrial conduction homogeneity by apelin. The protective effects of apelin from fibrotic remodeling were mediated by suppression of Smad2-dependent fibrosis.

**Conclusion:**

Apelin potently inhibited Ang II-induced atrial fibrosis and subsequent vulnerability to AF induction via suppression TGF-β/Smad2/α-SMA pathway. Our results indicated that apelin might be an effective up-stream therapy for atrial fibrosis and AF.

## Introduction

Atrial fibrillation (AF) is one of the most common arrhythmias, with high incidence rate and mortality. Atrial fibrosis is a typical manifestation of atrial structural remodeling, which promotes the occurrence and maintenance of AF, and significantly enhances the AF vulnerability (Andrade et al., 2014; Heijman et al., 2014). Angiotensin II (Ang II) can induce the proliferation of fibroblasts and the development of atrial fibrosis (Dzeshka et al., 2015; Hu et al., 2015).

Apelin is an endogenous peptide with strong positive inotropic effect. Apelin receptor shares 31% sequence identity with the human angiotensin type 1 (AT1) receptor, and the activation of the apelin receptor triggers various signaling pathways that exert protective cardiovascular effects (Marsault et al., 2019). More and more evidences show that apelin plays a protective role in the development of cardiovascular diseases (Cao et al., 2015; Zhong et al., 2017). Previous studies have found that apelin could inhibit the development of myocardial fibrosis (Huang et al., 2016; Xu et al., 2016). However, the effect and underlying mechanisms of apelin on AF and atrial fibrosis remain unclear.

## Materials and Methods

### Ethics Statement

All the research procedures and the use of laboratory animals were in accordance with the Guide for the Care and Use of Laboratory Animals (NIH Publication 2011, eighth edition), and were approved by the Animal Care and Use Committee of the Xinjiang Medical University. All animals were supplied with filtered water and standard laboratory diet. They were placed in a room with constant temperature of 23°C and 12 h light-dark cycle.

### Animal Model and Handling

48 male Sprague-Dawley (SD) rats (weighing 400–500 g, the Experimental Animal Center of Xinjiang Medical University, China) were randomly divided into three groups: control, Ang II and Ang II + apelin group. The osmotic mini-pumps were implanted subcutaneously (ALZET, Cupertino, CA, United States) in all animals for 2 weeks. Three groups were divided according to the different contents of osmotic mini-pumps as follows: Ang II group (Ang II, 1080 μg/kg/24h, Sigma-Aldrich, United States), Ang II + apelin group (Ang II 1080 μg/kg/24h, Sigma-Aldrich, United States and apelin 140 μg/kg/24h, Sigma-Aldrich, United States), control group (double distilled water). The dosage and the administration method of the drug were done according to the previous published studies (Scimia et al., 2012; Chagnon et al., 2017). Blood samples were collected at baseline and 2 weeks after drug administration in all the groups. Electrophysiological investigation were performed on all animals after 2 weeks of drugs administration. After that, the left atrial tissue was taken for staining and protein detection. All animal procedures were performed with ketamine anesthesia. Euthanasia was performed by intravascular pentobarbital overdose.

### Electrophysiological Investigation

Atrial stimulation was performed as previously described by Rudolph et al. (2010) In brief, a quadrupolar electrophysiological catheter (5F, Boston Scientific Corporation, United States) was inserted via the esophagus into the atrium. Electrophysiological recording and atrial stimulation were performed using LEAD-7000 Electrophysiology Management System (Jinjiang Co. Ltd., China). Atrial burst stimulation was performed at pacing stimulus amplitudes of 2.0 mA for 30 s at S1S1 stimulation cycle lengths starting at 50 ms with 10 ms stepwise reduction down to 10 ms. A recovery period of 30 s was maintained between the stimulation process. Atrial fibrillation is defined as the appearance of rapid, scattered atrial electrograms accompanied by irregular AV-nodal conduction and ventricular rhythm with a duration of these electrograms of 1 s. The AF inducibility (the numbers of induced AF in a certain period of time) and duration (defined as the first sinus-rhythm P wave after AF to the last stimulus-spike) were analyzed.

### Atrial Electrophysiological Mapping

The rats were placed on the operating table and connected to a ventilator. The thorax was opened and the heart was exposed. A 32-electrode microelectrode array (MEA, Multichannel Systems, Germany) was placed on the epicardial surface of the right atrium. Using a 128-channel, computer-assisted recording system to recorded electrograms. The inhomogeneity was reflected by the differences of activation time for each electrode with neighboring points, and the inhomogeneity index was obtained by calculating the largest difference at each electrode in the velocity-time curve according to the previous studies (Li et al., 1999; Shinagawa, 2002).

### Ang II and Apelin Assay

Plasma levels of Ang II and apelin were quantified with apelin-13 EIA Kits (Sigma-Aldrich, United States) and Ang II EIA Kits (Sigma-Aldrich, United States) according to the manufacturer’s instructions.

### Hematoxylin-Eosin (HE), Masson’s Trichrome and Picrosirius Red Staining

After the tissues of left atrial wall were obtained, it was immediately fixed with 4% paraformaldehyde at 4°C and then embedded in paraffin. Light microscopy was performed using semi-thin sections (2 μm) stained with HE and Masson’s trichrome stains. The Picrosirius Red staining was performed using 4 μm sections. After dewaxing, rehydrating and staining with Wiegert’s hematoxylin, the sections were stained in Picrosirius Red solution (0.5 g Sirius red F3B in 500 mL saturated aqueous picric acid solution) for 1 h. The sections were then washed in acidified water solution (5 mL acetic acid in 1 L water). Finally, sections were dehydrated in 100% ethanol, cleared in xylene and mounted in a resinous medium. Picrosirius Red stains the collagen red on a pale-yellow background in bright field microscope, and collagen appears bright orange-red. The area of myocardial fibrosis was measured by a digital imaging system (ImageJ, NIH Image, United States).

### Detection of Protein Expression by Western Blot Analysis

Membrane proteins were extracted from the tissue samples of the left atrium (LA) with 5 mmol/L Tris–HCl (pH 7.4), 2 mmol/L EDTA, 5 μg/mL leupeptin, 10 μg/mL benzamidine and 5 μg/mL soybean trypsin inhibitor. All procedures were performed at 4°C. Lysates were centrifuged at 10,000 *g* for 10 min, and supernatants (30 g protein/lane) were separated by SDS-PAGE on a 10% acrylamide gel. Gels were electroblotted onto a nitrocellulose membrane, then blocked with 2% non-fat dry milk and incubated with either anti-p-Smad-2 (Ser 465/467) (3108) and anti-Smad-2 (5339) (Cell Signaling Technology). The signals were scanned and semi quantitated using a digital imaging system (ImageJ, NIH Image, United States).

### Co-staining of Atrial Tissue With DDR2 (Tyrosine Kinase Receptor for Fibrillar Collagen) and α-SMA (Marker of Myofibroblasts)

DDR2 and α-SMA co-staining was performed on tissue sections to determine the extent of fibrosis. Sections were incubated overnight in mouse anti-rat DDR2 antibody (1:100 dilution, Sigma-Aldrich, United States) with rabbit anti-rat α-SMA (1:500 dilution, Sigma-Aldrich, United States) and then incubated with goat anti-rabbit Alexa Fluor^®^ 488 and goat anti-mouse Alexa Fluor^®^ 594-conjugated secondary antibodies at a dilution of 1: 5000 and mounted with VectaShield^®^ Antifade Mounting Medium with DAPI. Slides were visualized under fluorescent microscope (LeicaTCS SP8, Germany, atrial tissue, ×20).

### Statistical Analysis

Data entry and analysis were done using the IBM^®^ Statistical Package for the Social Sciences (SPSS) version 23. The normality of distribution of all the data were tested, and non-parametric tests were used if they were not normally distributed. The location and spread of continuous variables were presented as mean and standard deviation (SD) (if normal in distribution) or by the median (M) and inter-quartile range (skewed distribution). Hypothesis testing of the difference between the two groups of continuous variables was done using Student’s *t*-test (normal distribution) or the Mann–Whitney test (skewed data). Null hypothesis was rejected at *P* < 0.05.

## Results

**FIGURE 1:**
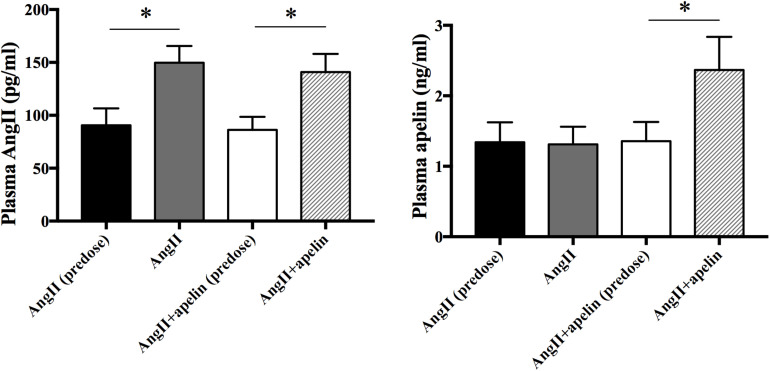
Plasma Ang II and apelin levels. Ang II plasma levels were significantly higher in both Ang II (*n* = 16) and Ang II + apelin groups (*n* = 16) compared with the corresponding groups before administration. Apelin plasma levels were significantly higher in Ang II + apelin group than predose. ^∗^*P* < 0.001.

To verify the release of Ang II and apelin from the osmotic pumps, Ang II and apelin plasma levels were measured before and 2 weeks after drug administration in all the groups. Ang II plasma levels were significantly higher in both Ang II and Ang II + apelin groups compared with the corresponding group before administration (149.69 ± 15.87 vs 90.54 ± 16.02 pg/mL, *P* < 0.001; 140.90 ± 17.10 vs 86.33 ± 12.25 pg/mL, *P* < 0.001; respectively). There was no significant difference was observed in the plasma levels between Ang II and Ang II + apelin groups (149.69 ± 15.87 vs 140.90 ± 17.10 pg/ml, *P* = 0.105). The plasma levle of Apelin in the Ang II + apelin group was significantly higher than the baseline (2.37 ± 0.47 vs 1.36 ± 0.27 ng/mL, *P* < 0.001), and no significant difference was observed before and after the drug administration in Ang II group (1.34 ± 0.28 vs 1.31 ± 0.25 ng/mL, *P* = 0.76) (Figure 1).

### Apelin Suppresses Atrial Fibrosis

**FIGURE 2:**
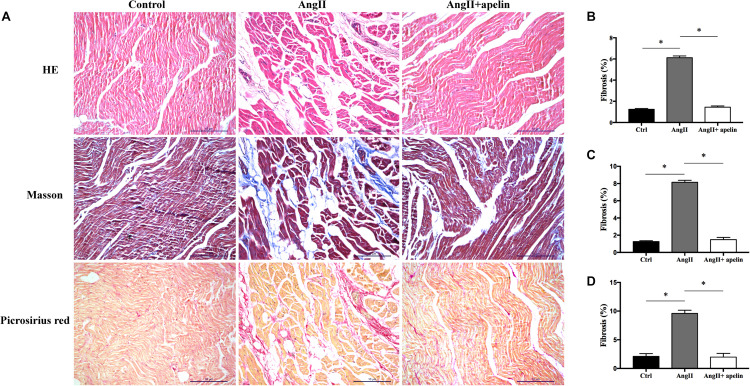
Atrial fibrosis of control, Ang II and Ang II + apelin groups (*n* = 16 in each group). **(A)** Representative images of HE, Masson’s trichrome staining and Picrosirius Red stained atrial sections. **(B–D)** Quantification of fibrotic areas of HE, Masson’s trichrome and Picrosirius Red stained atrial sections, respectively. Ang II group showed a higher extent of atrial fibrosis, while concomitant apelin treatment led to a significant reduction of atrial fibrotic areas in Ang II + apelin group. Scale bar indicates 10 μm. ^∗^*P* < 0.001.

Hematoxylin-Eosin, Masson’s trichrome, and Picrosirius Red stained atrial sections revealed significantly greater atrial fibrosis in the Ang II group compared with the control group (6.12 ± 0.16 vs 1.24 ± 0.09, *P* < 0.001; 8.15 ± 0.23 vs 1.25 ± 0.11, *P* < 0.001; 9.59 ± 0.56 vs 2.11 ± 0.47, *P* < 0.001, respectively). On the other hand, HE, Masson’s trichrome and Picrosirius Red stained atrial sections exhibited significant reduction in the levels of atrial fibrosis in Ang II + apelin group compared with Ang II group (1.45 ± 0.11 vs 6.12 ± 0.16, *P* < 0.001; 1.49 ± 0.25 vs 8.15 ± 0.23, *P* < 0.001; 1.98 ± 0.64 vs 9.59 ± 0.56, *P* < 0.001, respectively), and were in fact equivalent to the control group (1.45 ± 0.11 vs 1.24 ± 0.09, *P* = 0.08; 1.49 ± 0.25 vs 1.25 ± 0.11, *P* = 0.06; 1.98 ± 0.64 vs 2.11 ± 0.47, *P* = 0.42, respectively), (Figure 2).

### Apelin Preserves Atrial Conduction Homogeneity

**FIGURE 3:**
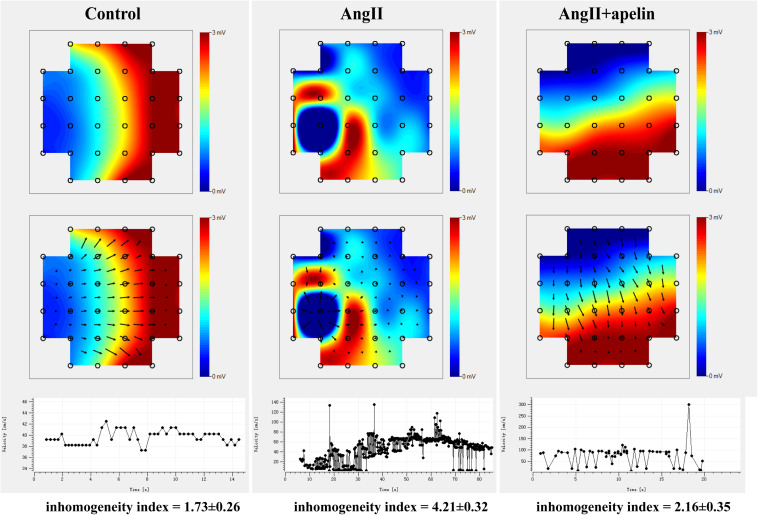
Representative images of epicardial multi-electrode activation mapping. The arrow indicates the conduct direction. The images of epicardial multi-electrode activation mapping and velocity-time curve both demonstrated significant inhomogeneity increase in Ang II group compared with control group (inhomogeneity index of Ang II group vs. control group = 4.21 ± 0.32 vs 1.73 ± 0.26, *P* < 0.001), which was completely abolished in Ang II + apelin group (inhomogeneity index of Ang II group vs Ang II + apelin group = 4.21 ± 0.32 vs 2.16 ± 0.35, *P* < 0.001) (*n* = 16 in each group).

Microelectrode array results demonstrated significant increase in the inhomogeneity of Ang II group compared to the with control group (inhomogeneity index of Ang II group vs control group = 4.21 ± 0.32 vs 1.73 ± 0.26, *P* < 0.001),but there was no significant increase in Ang II + apelin group (inhomogeneity index of Ang II group vs Ang II + apelin group = 4.21 ± 0.32 vs 2.16 ± 0.35, *P* < 0.001), (Figure 3 and Supplementary Videos 1–3).

### Apelin Inhibits Fibrosis by Inhibition of TGF-β1/SMAd2/α-SMA Pathway

**FIGURE 4:**
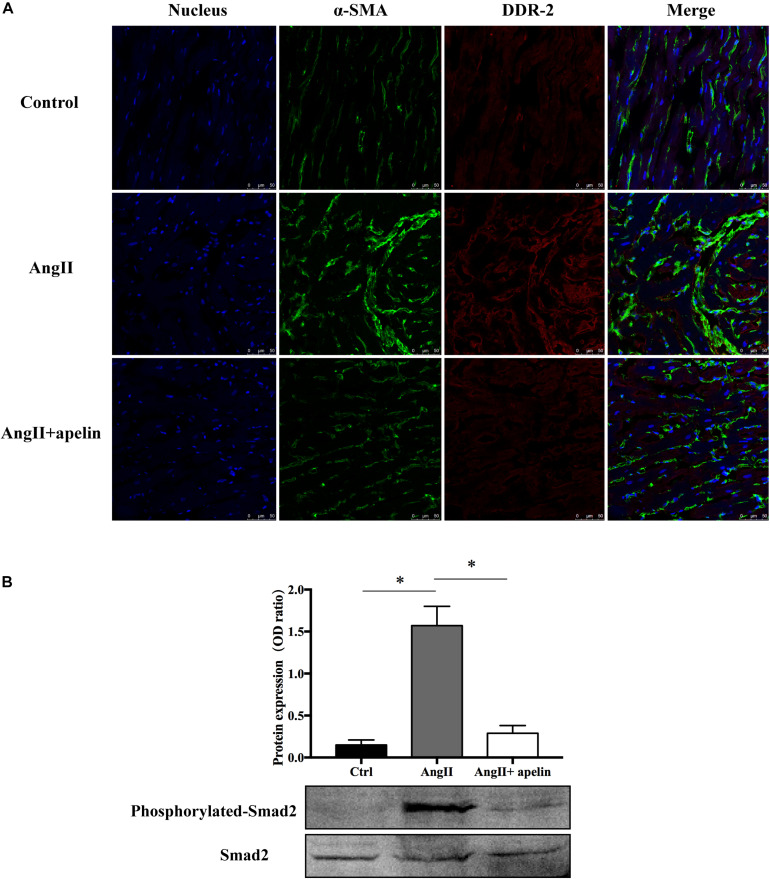
Apelin inhibits fibrosis by inhibiting TGF-β1/Smad2/α-SMA pathway. **(A)** DDR2/a-SMA co-staining of murine atrial sections showed a significant higher amount of fibrosis in the atrial tissue of Ang II group compared with the control group, while apelin treatment reduced the fibrosis to the level of animals in the control group. Scale bar indicates 50 μm. **(B)** Apelin reduces TGF-β1-induced phosphorylation of Smad2, as detected by western blot. ^∗^*P* < 0.001 (*n* = 16 in each group).

Co-staining of atrial tissues with DDR2 and α-SMA revealed a significant increase in the fibroblasts in Ang II group compared with the control group, whereas this effect was almost completely blunted in the Ang II + apelin group (Figure 4A).

Western blot analysis demonstrated that the Smad2 phosphorylation was significantly attenuated in the Ang II + apelin group compared to the Ang II group (0.29 ± 0.09 vs 1.57 ± 0.23, *P* < 0.001) (Figure 4B).

### Apelin Reduces AF Vulnerability

**FIGURE 5:**
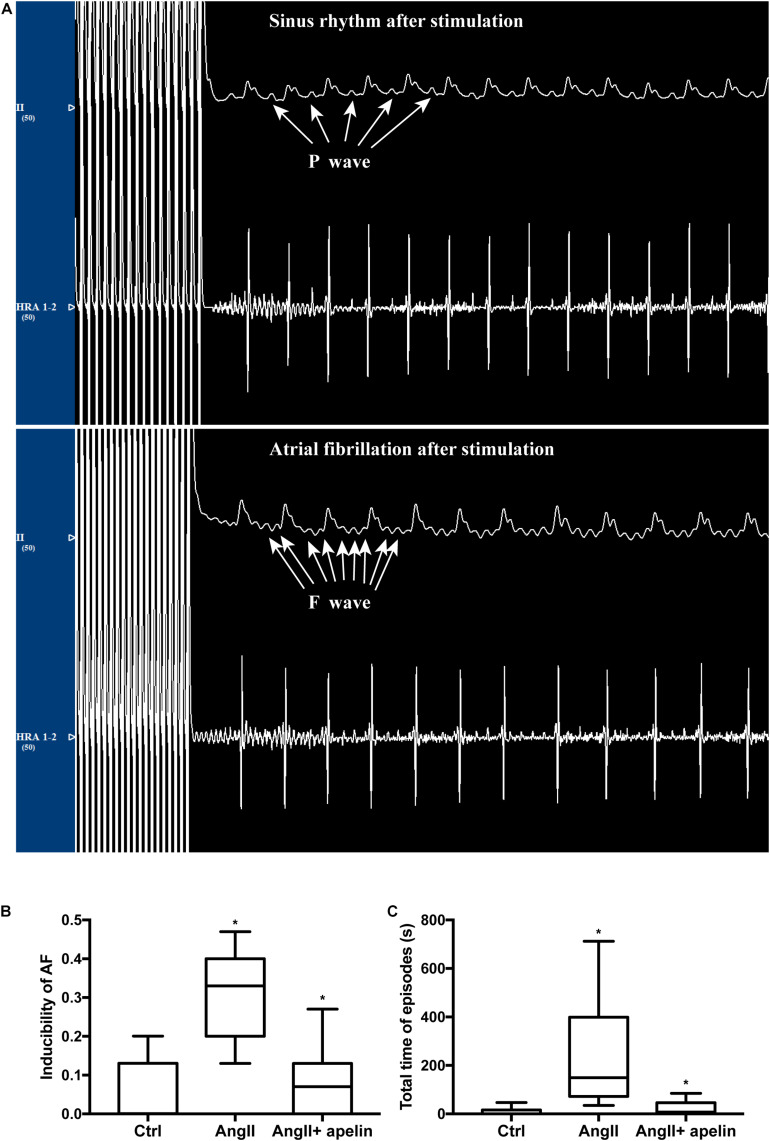
Apelin attenuatation elevated AF vulnerability. **(A)** Representative figures of ECGs with and without atrial fibrillation episodes after stimulation. ECG interference caused by stimulus current could not be ruled out. **(B,C)** Ang II treatment led to a distinct elevation of inducibility of AF and the total time of AF episodes, whereas in the Ang II + apelin group, the inducibility of AF and total time of AF episodes are significantly decreased. ^∗^*P* < 0.001 (*n* = 16 in each group).

The AF vulnerability (including inducibility of AF and AF duration) was significantly increased in Ang II group compared with the control group (inducibility of AF: *z* = −4.70, *P* < 0.001; AF duration: *z* = −4.746, *P* < 0.001) while the Ang II + apelin group demonstrated reduced AF vulnerability than Ang II group (inducibility of AF: z = −4.40, P < 0.001; total time of AF episodes: *z* = −4.349, *P* < 0.001), (Figure 5).

## Discussion

Atrial fibrillation is an arrhythmia that often leads to serious complications. Atrial remodeling includes electrical remodeling and structural remodeling, which are closely related to the occurrence and maintenance of AF. Atrial fibrosis is an important part of structural remodeling (Nattel and Harada, 2014; Opacic et al., 2016). In the present study, we observed that apelin administration suppressed atrial fibrosis as well as preserved atrial conduction homogeneity, and finally reduced AF vulnerability.

The expression of Ang II was increased in AF patients, and acted as a significant signaling mediator in the pathogenesis of atrial fibrosis (Dzeshka et al., 2015; Opacic et al., 2016). Rosin et al. (2013) demonstrated that Ang II could activate TGF-β1 synthesis, secretion, and downstream Smad2 signaling pathways, resulting in Smad2-dependent production of connective tissue growth factor. This in turn leads to the proliferation of fibroblast in the myocardium and promotes the differentiation of fibrocyte into myofibroblast, which eventually leads to the deposition of extracellular matrix (ECM). ACEI and ARB have been shown to block the activation of renin-angiotensin system (RAS) system, and block the atrial structural remodeling induced by Ang II, reducing the onset of AF. However, no study has reported the role of apelin in the inhibition of RAS system in AF.

In our study, apelin completely abolished the pro-fibrotic effects of Ang II treatment. Up to now, myofibroblasts are still the markers of pathological fibrosis and remodeling of myocardium. The process of differentiation from fibroblasts to myofibroblasts is crucial for promoting the deposition of ECM and increase the incidence of arrhythmia (Weber et al., 2013; Piek et al., 2016).

**FIGURE 6:**
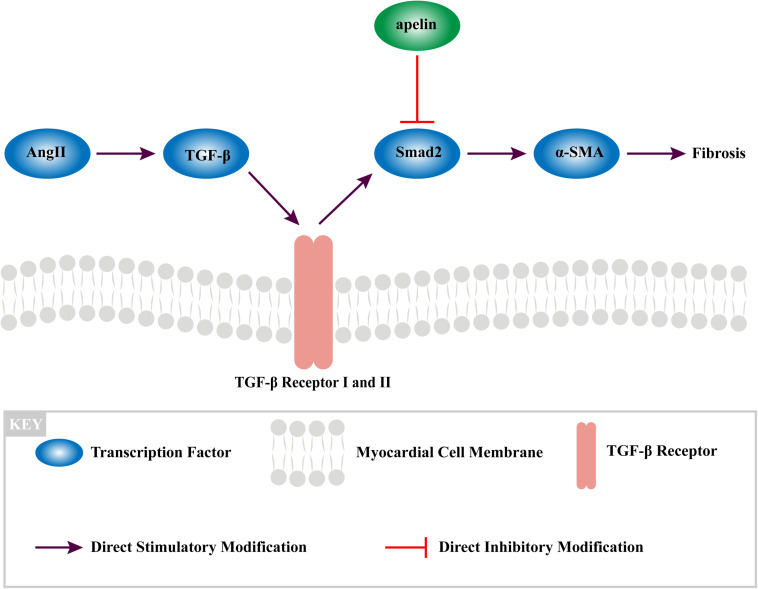
The mechanism of apelin reduces fibrosis in the atrium of Ang II-treated rat through TGF-β1/Smad2/α-SMA signaling pathway.

Furthermore, myofibroblasts also have the function of secreting angiotensin TGF-β1 and Ang II, which eventually leading to fibrosis.(Ghavami et al., 2015; Rudolph et al., 2015; Salvarani et al., 2017). Although it is not the only mechanism linking Ang II signaling and fibrosis, our study has found that Apelin could interrupt this vicious circle by mediating with Smad2-related suppression of myofibroblast differentiation, exerting the anti-fibrotic effect (Figure 6).

VALUE (Schmieder et al., 2008) and Val-HeFT (Maggioni et al., 2005) studies found that valsartan could reduce the incidence and recurrence rate of AF, but its underlying mechanism is unclear. In addition, Ellinor et al. (2006) have shown that valsartan could increase the plasma levels of apelin expression *in vivo*. It is unclear as to whether this effect of valsartan is achieved by the action of apelin. This is further confirmed by our present study results: valsartan increases the level of apelin *in vivo* and then inhibits Ang II-induced atrial fibrosis and subsequent vulnerability to AF. However, some studies (GISSI-AF Investigators et al., 2009) demonstrated that no significant improvement was observed in the prognosis of AF patients after using ARB drugs. The possible reason is that the beneficial influence of ARB is hard to work on the irreversible atrial remodeling.

Meanwhile, Parikh et al. (2013) found that the plasma Apelin level was significantly decreased in AF patients, which may be due to the disruption of apelin levels in atrial endocrine function during AF. If the above hypothesis is true, then apelin acts as a key factor, preventing the occurrence and development of AF. The decreased apelin levels in AF may be an underlying key factor that intervenes in the remodeling mechanisms leading to the phenomenon of “AF begets AF.” Therefore, based on the above theory, supplementation of apelin in AF cohort might disrupt the vicious cycle.

## Limitation

Our study has few limitations. Firstly, the mechanisms of AF are complex and unclear in clinical, we used a murine model of AF, and limitations occur when translating the present findings to human. Nevertheless, the key points of the present study were to evaluate the effect of apelin on atrial fibrosis and subsequent AF, for which the murine models have been widely used. Secondly, we did not evaluate the effect of apelin on Ang II-induced atrial remodeling in dose-dependent manner. In future studies, various doses of apelin should be taken into consideration to further elucidate whether the inhibitory effect of apelin on atrial remodeling was dose-dependent. Thirdly, although apelin can prevent fibrosis, the fibrosis may not be reversed when it already exists. Therefore, for patients with persistent AF who already have severe atrial fibrosis, whether apelin has ideal effect needs to be further verified.

## Clinical Application

Our findings suggested that apelin may be an effective up-stream treatment for atrial fibrosis. Further researches on the clinical use of apelin are necessary.

## Conclusion

In recent years, many scholars have carried out in-depth researches on the treatment of atrial fibrosis and AF (Adam et al., 2015; Chen et al., 2015; Henry et al., 2015; Jalife and Kaur, 2015). But up to now, there is no effective treatment to inhibit atrial fibrosis (Dzeshka et al., 2015; Van Wagoner et al., 2015; Lüscher, 2017), however, apelin may serve as an effective upstream therapy for this unmet clinical need. In the current study, we found that apelin potently inhibits Ang II-induced atrial fibrosis and subsequent vulnerability to AF via supression of TGF-β1/Smad2/α-SMA pathway.

## Data Availability Statement

The original contributions presented in the study are included in the article/Supplementary Material, further inquiries can be directed to the corresponding author/s.

## Ethics Statement

The use of animals and all procedures were in agreement with the Guide for the Care and Use of Laboratory Animals (NIH Publication 2011, eighth edition) and were approved by the Animal Care and Use Committee of the Xinjiang Medical University.

## Author Contributions

BT and XZ: work design. WL and LZ: experiments and data collection. XC, HW, and WQ: write the manuscript. All authors contributed to the article and approved the submitted version.

## Conflict of Interest

The authors declare that the research was conducted in the absence of any commercial or financial relationships that could be construed as a potential conflict of interest.
